# A Board Level Intervention to Develop Organisation-Wide Quality Improvement Strategies: Cost-Consequences Analysis in 15 Healthcare Organisations

**DOI:** 10.34172/ijhpm.2020.91

**Published:** 2020-06-28

**Authors:** Estela Capelas Barbosa, Lorelei Jones, Linda Pomeroy, Glenn Robert, Susan Burnett, Janet E. Anderson, Steve Morris, Fulop Naomi

**Affiliations:** ^1^Department of Applied Health Research, University College London, London, UK.; ^2^University of Bangor, Bangor, UK.; ^3^National Nursing Research Unit, Florence Nightingale School of Nursing and Midwifery, King’s College London, London, UK.; ^4^Centre for Patient Safety and Service Quality, Faculty of Medicine, Imperial College London, London, UK.; ^5^Florence Nightingale School of Nursing and Midwifery, King’s College London, London, UK.; ^6^Department of Public Health and Primary Care, University of Cambridge, Cambridge, UK.

**Keywords:** Quality Improvement, NHS, Cost-Consequence, United Kingdom

## Abstract

**Background:** Hospital boards have statutory responsibility for upholding the quality of care in their organisations. International research on quality in hospitals resulted in a research-based guide to help senior hospital leaders develop and implement quality improvement (QI) strategies, the QUASER Guide. Previous research has established a link between board practices and quality of care; however, to our knowledge, no board-level intervention has been evaluated in relation to its costs and consequences. The aim of this research was to evaluate these impacts when the QUASER Guide was implemented in an organisational development intervention (iQUASER).

**Methods:** We conducted a ‘before and after’ cost-consequences analysis (CCA), as part of a mixed methods evaluation. The analysis combined qualitative data collected from 66 interviews, 60 hours of board meeting observations and documents from 15 healthcare organisations, of which 6 took part on iQUASER, and included direct and opportunity costs associated with the intervention. The consequences focused on the development of an organisation-wide QI strategy, progress on addressing 8 dimensions of QI (the QUASER challenges), how organisations compared to benchmarks, engagement with the intervention and progress in the implementation of a QI project.

**Results:** We found that participating organisations made greater progress in developing an organisation-wide QI strategy and became more similar to the high-performing benchmark than the comparators. However, progress in addressing all 8 QUASER challenges was only observed in one organisation. Stronger engagement with the intervention was associated with the implementation of a QI project. On average, iQUASER costed £23 496 per participating organisation, of which approximately 44% were staff time costs. Organisations that engaged less with the intervention had lower than average costs (£21 267 per organisation), but also failed to implement an organisation-wide QI project.

**Conclusion:** We found a positive association between level of engagement with the intervention, development of an organisation-wide QI strategy and the implementation of an organisation-wide QI project. Support from the board, particularly the chair and chief executive, for participation in the intervention, is important for organisations to accrue most benefit. A board-level intervention for QI, such as iQUASER, is relatively inexpensive as a proportion of an organisation’s budget.

## Background

**Key Messages**

** Implications for policy makers**

- Board-level quality improvement (QI) interventions like iQUASER may be effective for the development of an organisation-wide QI strategy.
- Support from the chair and chief executive and board more widely is important for organisations to accrue most benefit from board-level QI interventions.
- Compared to other QI initiatives, a board-level intervention for QI, such as iQUASER, is relatively inexpensive as a proportion of an organisation’s budget.

** Implications for the public**

Several healthcare organisations take some form of action to improve quality for patients in their care. Quality improvement (QI) initiatives can range from specific focused small projects to bigger endeavours that involve several layers of management in the organisation. iQUASER was a board-level QI intervention to help senior hospital leaders develop and implement QI strategies. We have found organisations who took part in iQUASER made greater progress in developing an organisation-wide QI strategy and became more similar to the high-performing benchmark. We also found that stronger engagement with the intervention was associated with the implementation of a QI project. On average, iQUASER costed £23 496 per participating organisation, which is relatively inexpensive as a proportion of their budget.

The Francis inquiry into serious failings of care at Mid Staffordshire National Health Service (NHS) Trust highlighted the need for hospitals and other healthcare organisations to have quality improvement (QI) strategies.^1^ As a result, national level regulators are increasingly concerned with supporting the boards of healthcare organisations to devise strategies for QI.^2^ In England, healthcare organisations may incorporate more than one hospital, overseen by a single corporate board and are characterised by type of service provided (acute, community or mental health), foundation trust status^[1]^, performance status (as assessed by the English healthcare regulator) and number and location of sites.

Previous research has shown that the boards of healthcare organisations have an important role in leading and overseeing quality and safety.^6,7^ However, there are few studies of board-level interventions, their impact, or associated costs. In this paper, we report the costs and consequences from an evaluation of a board-level organisational development intervention (iQUASER).

The intervention was based on a guide to assist senior hospital leaders to develop an organization-wide QI strategy. This guide was based on findings from the QUASER study, a collaboration between 5 European countries (England, Norway, Sweden, the Netherlands, Portugal) funded by the European Union FP7 programme.^8^ For the intervention, Foresight Partnership (FP) (an organizational development consultancy now part of GE Healthcare) facilitated the use of the guide in 6 NHS healthcare organisations between July 2014 and May 2015. We studied the impact of this intervention using a mixed-method approach, which included a cost-consequences analysis (CCA), which computes incremental costs and compares it to some of the consequences of the intervention. We also carried out an in-depth qualitative process evaluation, which we have reported elsewhere.^9^ In the present paper, we report findings from the cost-consequence analysis.

###  The QUASER Guide

The QUASER Guide (https://www.eur.nl/sites/corporate/files/QUASER-GuideForHospitals_0) was designed to help senior leaders (boards) of healthcare organisations, wherever they are on the ‘quality journey,’^10^ as a dialogical tool,^11^ to reflect on, develop, and implement organisation-wide QI strategies. It was based on detailed research conducted in hospitals in 5 European countries during the period April 2011-March 2012 and included consideration of the national healthcare context in each of the participating countries. In total, 387 interviews and 796 hours of observation (including of 176 meetings relating to QI) were undertaken.^8^

**Figure 1:**
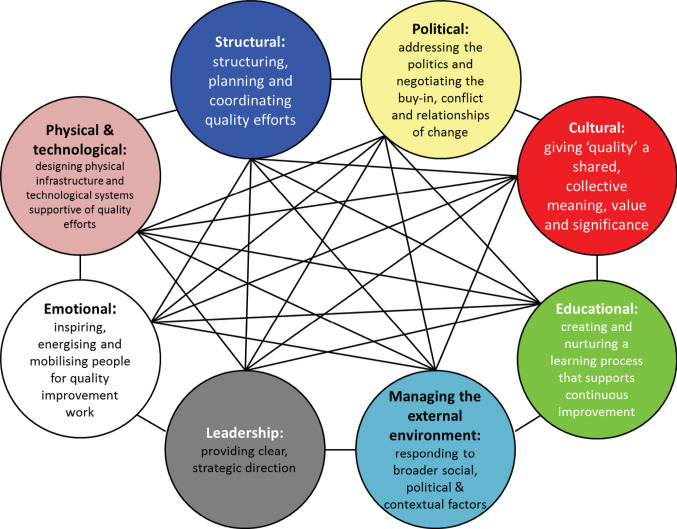
Eight Challenges Highlighted in the QUASER Guide.

The guide is structured around 8 interlinked challenges for QI, as shown in Figure 1. These are: Leadership, Political, Cultural, Educational, Emotional, Physical and Technological, Structural, and External Demands. Drawing from the QUASER research findings, the guide also provides some suggested *strategies* for how healthcare organisations could better deliver high quality and safe services. It then provides *examples* from hospitals that have already implemented these strategies elsewhere in Europe.

###  The iQUASER “Intervention”

The aim of the intervention was touse the QUASER Guide to help senior leaders (boards) of organisations develop their organisation-wide QI strategies. A full list of senior leaders who took part on the intervention is available in Supplementary file 1. The intervention was developed and delivered by an organisational development consultancy, FP, based on the dialogical principles of the guide. Each healthcare organisation paid a fee of £5000 to participate in the iQUASER intervention. They were also offered a tailored and intensive consultancy service at a higher fee, but all organisations chose the lower cost option. The intervention consisted of 4 phases over a 10-month period (July 2014-April 2015). The intervention has been described in detail in Supplementary file 2, but in short Phase 1 consisted of the sign up and introduction to the programme, which was presented at a Board Meeting. Phase 2 involved the completion of an organisational self-assessment tool based on the QUASER Guide’s 8 challenges. Participants (board members) completed an online questionnaire to assess how their organisation had addressed each of the 8 QUASER challenges. From the results of the self-completed assessment tool, FP generated a report for each healthcare organisation to discuss and build a shared view of the key QI challenges for each organisation; each organisation was also asked to identify one specific organisation-wide QI project. Phase 3 comprised a workshop attended by senior leaders from the participating organisations. The objective of the workshop was to explore and develop approaches to implementing an organisation-wide QI strategy. Participants were encouraged to have arrived at specific goals or commitments to take their QI strategy forward. Finally, phase 4 consisted of 3 follow-up facilitatedaction learning sets, which explored implementation challenges, and ways to overcome them, as well as following up on progress on development of the organisation-wide QI strategy and the one selected organisation-wide QI project. In addition, in the final learning set participants were asked to reflect on the value of the guide in facilitating QI, as well as the value of facilitated support for implementation.

## Methods

###  Study Design

The evaluation of the iQUASER intervention comprised a mixed-methods ‘before and after’ study with 2 elements, an in-depth qualitative process evaluation and a CCA. The process evaluation explored how the intervention worked and described and explained the variation in the organisational response to the intervention, in order to distil learning for future interventions. The findings from this component are reported elsewhere.^9^ This paper presents findings from the CCA, in which qualitative data were used to define the consequences and the level of engagement of each organisation with the intervention. We have chosen to have the consequences derived from the qualitative part of the evaluation in line with a mix-methods approach.

 Board meetings attended by the research team were held in public, and organisations were informed of the presence of the research team, and this research, beforehand.

###  Sampling Criteria

**Figure 2:**
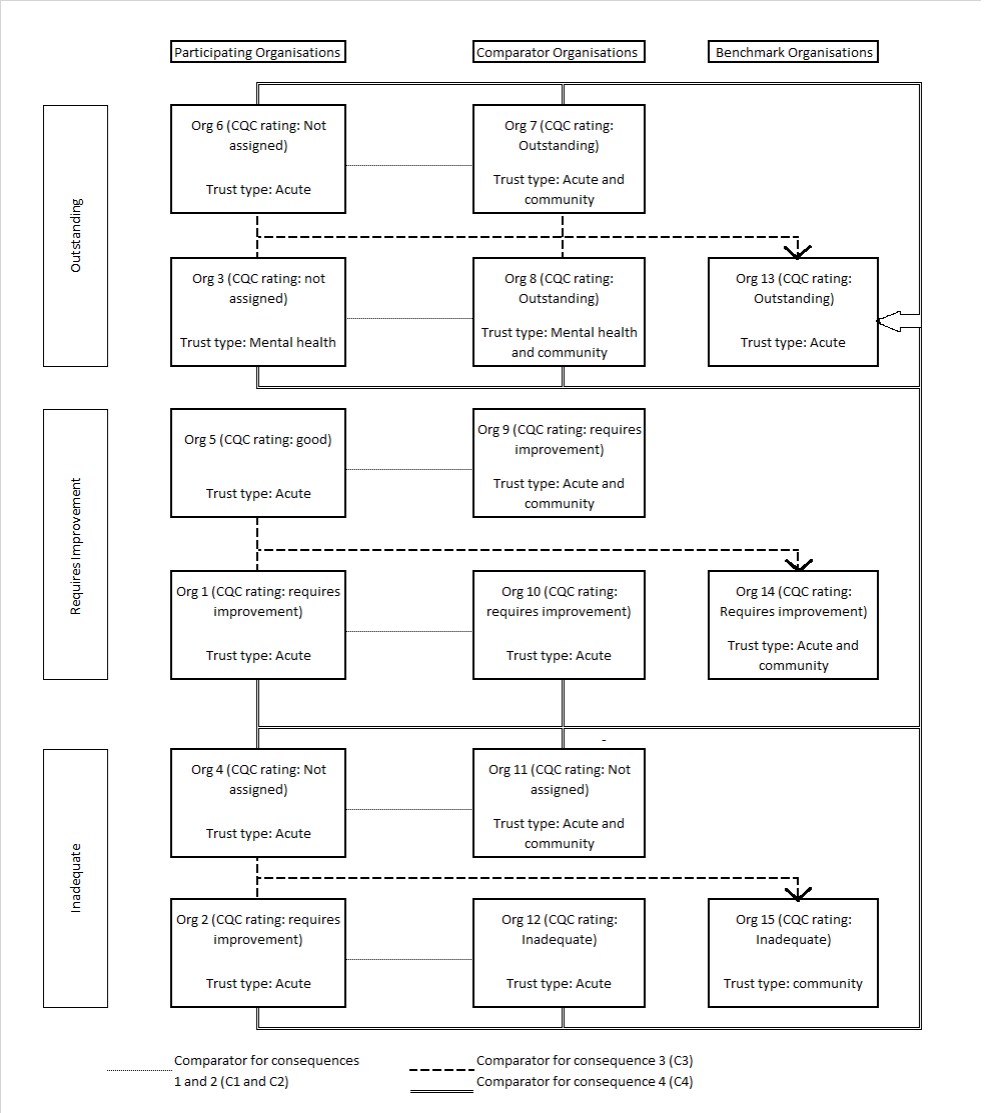
Participating Organisations, Comparators and Benchmarks. Abbreviation: CQC, Care Quality Commission.

A letter inviting hospital-sector organisations to participate in the intervention was sent to one of the executive directors of 9 healthcare organisations in a network of providers in a large urban area. These 9 organisations were selected due to their geographical location, as attendance to the workshops was a mandatory component of the intervention. Six organisations (5 acute care providers and 1 mental health provider) agreed to participate (the ‘participating organisations’). In England, healthcare provider organisations may incorporate more than 1 hospital, overseen by a single corporate board. Our study focused on organisations as the unit of analysis, and not individual hospitals. Data were also collected from a comparator group (‘comparator organisations’) of 6 organisations which individually matched each of those in the intervention group on the following dimensions: type of service provided (acute, community or mental health), foundation trust status^[1]^, performance (as assessed by the English healthcare regulator) and number and location of sites. We also selected an additional 3 organisations as benchmarks to reflect different performance levels (‘outstanding,’ ‘good’ and ‘requires improvement’ as rated by the English healthcare regulator, the Care Quality Commission, CQC), giving a total of 15 organisations. There were, therefore, three different groups of organisations: (1) those that participated in the intervention, named participating or intervention organisations (n = 6); (2) those matched to the participating organisations for comparison, called comparator organisations (n = 6) and (3) the benchmark organisations, which are compared to both the intervention and comparator organisations. Figure 2 show the possible comparisons.

###  Data Collection

Data were collected across 4 phases in all 15 organisations, and grouped into 3 time-periods – ‘before’ the intervention (March 2014–June 2014), ‘during’ the intervention (July 2014–April 2015) and ‘after’ the intervention (May 2015–November 2016). Interviews were undertaken with board members (4 to 6 per organisation) in the 6 organisations that had agreed to participate in the evaluation and in one comparator and one benchmark organisation which volunteered to take part in the interviews (interviews were not requested with the other organisations). In total, 37 interviews were carried out in 2014 and 28 in 2015/2016. We asked boards to nominate members, both executive and non-executive directors, including the non-executive director responsible for quality. Interviews were semi-structured and covered topics in relation to the governance of QI (Supplementary file 3). We also observed the public part of the board meeting on 2 occasions for all 15 organisations. In sum, we observed 60 hours of board meetings taking notes of quality-related discussions, who raised and discussed these issues, which of the 8 QUASER challenges they related to, time spent discussing quality and how quality-related issues were considered within each organisation. In addition, we collated relevant QI documentation. We used publicly available documents for the comparator and benchmark organisations and asked the 6 participating organisations to provide documents that most closely described their approach to QI. A range of other publicly available documents were also collected for analysis including: Quality Accounts^[2]^ for the last year; rolling 5-year strategy; board of directors’ minutes and other board meeting papers; Quality committee meeting minutes for the previous 3 months; CQC documents; and, any other relevant documents provided by the participating organisations.

###  Cost-Consequences Analysis

We undertook a CCA of the iQUASER intervention. This is a form of economic evaluation comparing alternative options in which the components of incremental costs (direct or indirect) and consequences (eg, knowledge, behaviours, and processes) are computed and listed, without aggregating the results into a cost-effectiveness ratio. This form of economic evaluation is distinct from both cost-effectiveness analysis and cost-benefit analysis, which tend to focus on a single outcome measure and aggregate costs and the outcome into a single summary measure.^12,13^ The CCA is the appropriate form of economic analysis to use in the present study given the range of possible outcome measures for the iQUASER intervention.

###  Measuring Consequences

In total, 6 quantifiable outcome measures were included, based on the literature and discussions within the team and with our FP partners^10,15-18^ (Table 1).

**Table 1:** Scale and Criteria for Measuring Consequences

**Measure**	**Consequence**	**Scale**
C1	Organisation-wide strategy	Very fragmented Moderately fragmented Managed internally One overall QI strategy
C2	Extent of addressing each of the 8 challenges	No evidence of awareness Planning Doing Evaluating an action undertaken Revision and wider learning
C3	Comparison with 'benchmark' Trust	Not very similar to the benchmark Similar to a small extent Similar to a moderate extent Similar to a large extent
C4	Comparison with high performing 'benchmark' Trust	Not very similar Similar to a small extent Similar to a moderate extent Similar to a large extent
C5	Engagement with the intervention	Minimal Moderate Strong
C6	Implementation	Not at all To a little extent To a moderate extent To a great extent

Abbreviation: QI, quality improvement. Note: For high-performing participating and comparator organisations, C3 and C4 were effectively the same because only one outstanding benchmark Trust was recruited (See Supplementary file 1, Tables S2 and S3).

 Developing and assigning the ratings for each outcome was an iterative, interpretive process that drew on qualitative data (interview accounts, observations of board meetings and documents), as well as team discussion. Initially members of the research team (LP, NF, JA, GR, SB) were assigned 3 organisations each to independently score them for each of the 6 measures described above based on a review of board minutes, board observations and quality documents. This initial process was carried out before September 2015 to ensure the ‘before’ ratings were unbiased. The research team then came together to discuss the initial ratings and agree on the definitions of the rating criteria. Once the definitions had been agreed, LP reviewed all ratings. The team met once again and agreed final scores (after September 2015). This process was repeated for every organisation and for each of the 6 attributes described below.

####  Organisation-Wide Strategy (C1)

 In order to assess the existence or development of an organisation-wide strategy (labelled consequence C1), we asked all participating organisations to provide us with the documents that represented their QI strategy and we triangulated these with documents previously collected (quality accounts, quality committee minutes, board meeting papers).

####  Addressing the QUASER 8 Challenges (C2)

To assess the extent to which organisations had jointly met each of the 8 challenges (labelled consequence C2), we analysed board meeting observations, minutes and the quality accounts ‘before’ and ‘after’ the intervention. We used the ‘quality cycle’^18^ to understand where in the QI journey each organisation was before and after the intervention, creating a 5-point scale (see Table 1, C2). For example, a healthcare organisation might be ‘planning’ to address the cultural challenge but had not yet done so; another organisation might have planned and carried out actions in relation to the physical challenge, and so forth.

####  Comparison With “Benchmark” Organisations

We wanted to understand how participating and comparator organisations compared to benchmarks. Therefore, a rating of how each intervention and comparator organisations compared with a ‘benchmark’ organisation was assigned based on documents and board observations and using the above consequences (C1, C2). Figure 2 shows the possible comparisons between organisations. While there were 3 benchmark organisations, we defined 2 different benchmarks comparisons: one that had been rated by the English healthcare regulator at an equivalent CQC level (this comparison was labelled consequence C3) or comparison with a high-performing ‘outstanding CQC rated’ benchmark (labelled consequence C4). Assessment of each (participating and comparator) organisation in relation to benchmark organisations was also based on a 4-point scale, defined as (1) not very similar to the benchmark, (2) similar to a small extent, (3) similar to a moderate extent, and (4) similar to a large extent. Intervention and comparator organisations were considered very similar to the benchmark based on how they compared in terms of their ratings for C1 and C2. We assumed that becoming more similar to the benchmark is an improvement, that is, a higher score in the scale in the after period is an improvement. This is an assumption, and while it seems logic particularly for the comparison with the ‘outstanding CQC rated’ benchmark, it could be argued that it is a deterioration when comparing to the ‘inadequate CQC rated’ benchmark. For consistency, we have always assumed that the more similar an organisation is to the benchmarks, the better. Assessments were made before and after the iQUASER intervention (March 2014/June 2014 and May 2015/November 2016, respectively). If an organisation had improved in C3 or C4 over time, this effectively meant that it had become more similar to the benchmark.

####  Engagement (C5)

 Engagement with the intervention (minimal, moderate and strong) was assessed on the basis of attendance at workshop/action learning sets, consistency of participants, seniority of participants.

####  Implementation of an Organisation-Wide QI Project (C6)

 Finally, assessment of the extent of implementation of the proposed organisation-wide QI project selected to be carried out during the iQUASER intervention was based on interviews with implementation leads immediately after the second learning set, and at the 12 months follow up, and relevant documents (board meeting papers, annual reports, slide packs). Implementation was rated according to the following scale: (1) none (eg, no actions had been taken); (2) to a little extent (eg, evidence of continued discussion and planning); (3) to a moderate extent (eg, evidence of some action); (4) to a great extent (eg, evidence of implementation).

####  Local Context During the Time of the Intervention

 The iQUASER intervention took place between July 2014 and April 2015. Considering that part of the evaluation was conducted before and after the intervention period, one could expand the time relevant to the intervention to fiscal years 2014/2015 and 2015/2016. These 2 fiscal years coincide exactly with the beginning of the coalition government, which according to the King’s Fund found additional funding in 2014/2015, both new, but mostly reallocated from within existing budgets, to support direct patient care within the NHS. The coalition government had also announced plans to increase the NHS budget by more than £3 billion in cash terms in 2015/2016, which was perceived as a change in the austerity trend that had imposed NHS salaries freeze in 2010/2011. Having said that, looking back retrospectively, it is understood that the NHS has been under severe financial pressure since 2010/2011 and that the coalition measures of 2014/2015 and 2015/2016 did not alleviate the austerity climate within the healthcare service.

###  Measuring Costs

We identified 7 cost components: (*a*) sign up to the intervention (upfront cost); (*b*) project engagement and presentation; (*c*) completion of self-assessment tool; (*d*) workshop; (*e*) team discussion; (*f*) follow up action learning sets; and (*g*) costs of activities and actions to meet workshop objectives. Although it was expected that iQUASER could result in an increase in the number of actions and interventions to improve health outcomes or reduce harm, we have explicitly chosen not to include the costs of these consequent actions and interventions, as these were downstream outputs and potential cost savings, which would be too diffuse to identify.

All costs were valued in monetary terms. These included (1) financial outlays that were incurred directly as a result of the iQUASER intervention, and (2) time costs incurred by staff participating in the intervention, which were valued in money terms using market prices from published unit costs.^19^ We took an NHS perspective, which meant we also included costs that were not borne by the participating organisations but would have been incurred by the NHS, eg, developing and delivering iQUASER and venue hire.

All costs refer to direct or opportunity costs incurred between the point of project sign up (July 2014) and the last learning set of the intervention (April 2015). For the time cost components, the research team, who were present at the presentation and discussion meetings, workshop and action learning sets, directly observed actual time spent on each activity (*b-f*). For cost component (*g*), an estimate of time was directly asked to participating organisations, and included only staff costs. All costs were measured in Great British Pounds (£) of 2015/2016.

## Results

###  Implementation of an Organisation -Wide QI Project 

 Following the intervention, 2 of the 6 participating organisations appointed a director of QI who was responsible for producing an organisation-wide QI strategy, and to instigate and coordinate QI activities across their organisations. These 2 organisations also implemented an organisation-wide QI project as intended (Organisation 6 set up an organisation-wide QI facility and organisation 5 implemented an organisation wide QI project for patients with diabetes). Organisation 5 did not produce an organisation-wide QI strategy but did implement a range of QI projects (a review of QI governance, introducing a smoking ban, and reducing the number of patients awaiting a bed and non-clinical transfers). The remaining organisations did not produce an organisation-wide QI strategy or implement an organisation wide QI project.

###  Engagement and Organisation -Wide Strategy

There appeared to be a positive relationship between the level of engagement with iQUASER, the development of an organisation-wide QI strategy, and the degree of implementation of a QI project. Table 2 shows that organisations that engaged moderately or strongly with the intervention were also the ones to improve in the development of an organisation-wide QI strategy. Only organisations that strongly engaged with iQUASER also implemented their proposed QI project to a large extent, whereas the ones that engaged minimally did not implement their proposed project.

**Table 2:** Level of Engagement and Degree of Implementation of the Intervention (Participating Organisations Only)

	**Level of Engagement **	**Organisation-Wide QI Strategy Improvement**	**Degree of Implementation of QI Project**
Organisation 1	Minimal	Not improved	Not at all
Organisation 2	Minimal	Not improved	Not at all
Organisation 3	Moderate	Improved	Moderate extent
Organisation 4	Moderate	Improved	Not at all
Organisation 5	Strong	Improved	Large Extent
Organisation 6	Strong	Improved	Large Extent

Abbreviation: QI, quality improvement.

 In other words, the 3 organisations that went on to implement an organisation-wide QI project, to some degree, following the intervention were more highly engaged with the intervention (as reflected in the consistency of participation, the seniority of board members who participated, and support from the Board for the intervention) than the 3 organisations that did not implement a QI project.

Our findings also suggest that support from the board, especially the chief executive officer (CEO) and chair, for participation in the intervention, was related to engagement with the intervention. For example, in Organisation 6, the CEO showed strong support for the intervention, attending all sessions (see Supplementary file 1, Table S1). In contrast, in Organisation 4, participation in the intervention was not supported by the board, indeed the chair was actively hostile to the intervention.

###  Improvements in Consequences C1-C4

Table 3 presents the results in terms of improvement in each outcome for participating and comparator organisations. Improvement was measured by comparing ratings before and after the iQUASER intervention. For organisation-wide QI strategy (C1), healthcare organisations that participated in iQUASER improved in the development of an organisation-wide strategy more than comparator organisations, that is, 4 of the total 6 participating organisations achieved an improvement, while only 2 out of 6 comparator organisations improved by one point or more. Addressing each of the 8 challenges (C2) was the consequence with the least observed improvement, with only one participating organisation improving by one or more points in the 5-point scale and no comparator organisation improving.

**Table 3:** Number of Organisations That Have Achieved an Improvement in Consequences (Before and After Improvement) – Participating and Comparators

**Consequences**	**Participating Organisations**	**Comparator Organisations**
Organisation-wide strategy (C1)	4	2
Extent of addressing each challenge (C2)	1	0
Comparison with ‘equally CQC rated benchmark' (C3)	4	2
Comparison with high-performing benchmark (C4)	6	2

Note: Tables S2 and S3 in Supplementary file 1 show the results for each organisation (participating, comparators and benchmarks) before and after the intervention respectively.

 More intervention organisations became more similar to their own CQC-rated benchmark (C3) than comparator organisations, with 4 of the total 6 participating organisations achieving an improvement, compared to only 2 out of 6 comparator organisations. Finally, all organisations that participated in iQUASER became more similar to the high-performing benchmark (C4), while only 2 comparator organisations moved towards this benchmark.

As the level of engagement with the intervention and improvements in the development of a QI strategy move in the same direction, we have chosen to look at the consequences by level of engagement. Table 4 presents the number of organisations (participating and comparators), where an improvement was observed after the intervention for consequences 1 to 4 (C1–C4), by level of engagement.

**Table 4:** Number of Organisations That Have Achieved an Improvement, by Level of Engagement

**Consequence**	**Trust Type**	**Number of Organisations**	**Organisation-Wide Strategy (C1)**	**Extent of Addressing Each Challenge (C2)**	**Comparison With Benchmark Trust (C3)**	**Comparison With High-Performing Benchmark Trust (C4)**
Strongly engaged	Participating	2	2	1	2	2
	Comparator	2	0	0	0	0
Moderately engaged	Participating	2	2	0	0	2
	Comparator	2	0	0	1	1
Minimally engaged	Participating	2	0	0	2	2
	Comparator	2	1	0	1	1

 We found that organisations that engaged strongly or moderately with the intervention improved by at least one point in the scale in terms of the development of an organisation-wide strategy (C1), whilst for those that only engaged minimally no improvement was observed. Comparator organisations matched to those that engaged moderately or strongly were less likely to achieve an improvement, with only 1 out of 6 actually improving by 1 point or more. Nonetheless, whilst no minimally engaged participating organisation improved in consequence one, one of the matched comparators has achieved an improvement in terms of the development of an organisation-wide QI strategy.

 In relation to the extent of addressing each of the 8 challenges (C2), in the participating group, only one organisation which strongly engaged with the intervention improved by one point or more. None of the comparator organisations improved by one point or more in this consequence.

 Thirdly, we compared the intervention and comparison organisations to the ‘benchmarks’: whilst all participating organisations became more similar to the high-performing ‘benchmark’ (C4) regardless of their level of engagement with the intervention, the same was not observed when comparing to their own CQC-level benchmark (C3). In the latter case, moderately engaged organisations have not become more similar to their benchmark. This could be because the 3 benchmarks had not improved their own performance. In fact, the high-performing benchmark organisation had decreased its performance in terms of addressing each of the 8 challenges, which may explain why participating organisations became more similar to this benchmark (C4). Finally, results show that fewer of the comparator organisations became more similar to their own CQC level benchmark (C3) and the high-performing one (C4), when compared to intervention organisations. In fact, only 2 out of 6 organisations have improved by one point in the defined scale in either case.

###  Cost Analysis

Table 5 presents the costs associated with the iQUASER intervention; the mean total cost per participating organisation was £23 496. The most costly component of the intervention was the workshop, which included the costs of developing and delivering the workshop itself and the staff time costs of having 3 to 5 board members attending a full day of activities. The second most costly component was the up-front cost of signing up to iQUASER. The third most costly component were the activities and actions to meet workshop objectives. This last component produced the greatest variation amongst participating organisations.

**Table 5:** Average Costs of the iQUASER Intervention Per Organisation, by Level of Engagement (in 2014/2015 UK Pounds)

**Component of Intervention**	**Strongly Engaged **	**Moderately Engaged **	**Minimally Engaged **	**Overall Mean**	**% Of Total Cost**
Sign up (upfront cost)	£5000	£5000	£5000	£5000	21.3%
Project engagement and presentation	£1175	£810	£960	£982	4.2%
Completing self-assessment	£1215	£1018	£828	£1021	4.3%
Workshop	£11 897	£11104	£11538	£11513	49.0%
Team discussion	£219	£323	£537	£360	1.5%
Follow up learning sets	£2080	£1536	£1536	£1717	7.3%
Activities and actions to meet workshop objectives	£2604	£5239	£868	£2904	12.4%
**Total**	**£24 190 **	**£25 029 **	**£21 267 **	**£23 496 **	**100%**
Total staff time across all components combined (in hours)	87.8	92.5	64.5	81.6	-
Monetised staff time costs across all components combined as a proportion of total costs	45.4%	47.2%	37.8%	43.7%	-
Number of organisations	2	2	2	Total	6

 An analysis of costs by level of engagement found that a higher level of engagement was associated with higher costs of project engagement and presentation, completing the self-assessment tool and, to a minor extent, participating in the follow up learning sets. The opposite was observed with regards to team discussion, component for which stronger engagement is associated with lower costs. Finally, whilst minimally engaged organisations appear to have a significantly lower overall cost, both moderately and strongly engaged organisations have a higher average cost when compared to the overall mean.

## Discussion

We have carried out an analysis of costs and consequences of a board level intervention to develop organisation-wide QI strategies in 15 hospitals in England. Our findings are consistent with those of the process evaluation, which found that organisational response was contingent on the level of engagement from the CEO and Chair, organisational ‘readiness,’ and the availability of ‘slack.’^9^

The cost consequence analysis has shown that organisations which moderately or strongly engaged with the intervention were more likely to have achieved an improvement in the development of an organisation-wide QI strategy and implemented their chosen QI project to a greater extent than less strongly engaged organisations. The level of engagement was defined by the attendance at workshop/action learning sets, consistency of participants, seniority of participants. This relationship between engagement in the intervention and implementation of an organisation-wide QI project could be interpreted as evidence that board-level interventions may facilitate organisation-wide QI. Support from the board, especially from the Chair and CEO for participation in the intervention, appeared to be particularly important. This supports other findings that commitment from senior management is fundamental in supporting organisational ‘absorptive capacity’ ie, the capacity to acquire, assimilate and apply knowledge to improve performance.^20^

Further, the finding that all organisations which participated in iQUASER became more similar to the high-performing benchmark organisation seems to suggest that board-level interventions, such as iQUASER, are one possibility for improving overall performance. This is in line with findings of the Leadership Saves Lives study,^21^ which also found that “authentic” participation from staff in QI initiatives was linked to substantial culture change. Other studies have also found that support from senior leadership is important even in other sectors, such as nursing and care homes.^22,23^

 However, our findings also have shown that minimally engaged organisations have also become more similar to the high-performing benchmark. Furthermore, a range of other QI initiatives took place in NHS organisations during the intervention time-period, including in the selected comparators, potentially confounding some of the results pointed out in our analysis. Therefore, our findings should be interpreted with care.

 Regarding the extent of addressing all 8 QUASER challenges, only one organisation achieved an improvement. This may reflect the difficulty in obtaining a multi-faceted improvement, or that organisations were attending to challenges according to the priorities at the time. Other possible explanations could be that the methods we used were unable to consistently detect changes regarding this consequence, or that organisations have prioritised developing a QI strategy rather than specifically addressing the QUASER challenges.

By considering staff time (opportunity) costs, we were able to estimate the cost of the intervention, which to our knowledge has rarely (if at all) been studied in the QI literature. The iQUASER intervention was relatively inexpensive as a proportion of total healthcare provider organisation’s yearly budgets. Finally, staff time costs were a relatively high proportion of the total costs. Thus, there may be a trade-off in terms of opportunity cost and capacity to benefit from the intervention, that is, for the intervention to be most beneficial, organisations may need to have capacity or ‘slack’ to engage.^9^

Although relatively inexpensive, iQUASER did not result in much improvement for the organisations that only minimally engaged with the intervention, raising questions about resource usage. It is not unusual for NHS organisations to invest in QI initiatives, but not devote the time and effort necessary to achieve a more substantive results.^2^ The relationship between level of engagement and costs can be partly explained by staff time inputs, as organisations that engaged more strongly with the intervention either devoted more of their time or involved more senior board members. In terms of time, the minimally engaged organisations devoted more than 20% less time to participating and obtaining outputs from iQUASER.

This analysis was innovative in that it has compiled both costs and consequences of a board-level QI intervention. One of the strengths of this analysis was that it performed several types of comparisons, including a direct comparison between intervention and comparator organisations, and the comparison of both intervention and comparator organisations with benchmarks in 2 different ways. Furthermore, the measured consequences were drawn from the qualitative analysis of iQUASER, making our analysis a true mixed-method study. The analysis of this paper was limited by the small number of participating healthcare provider organisations, by the lack of direct comparability between organisations, moderately overcome by the matched comparators. By using a mixed methods approach, we have effectively produced an analysis that uses both quantitative and qualitative measures. While we understand some subjectivity may rise from such approach, this is a common problem for organisational level interventions and our approach was to try and capture multiple aspects of the organisational response, which could inform other studies. In order to minimise this subjectivity, we studied QI using multiple data sources, triangulated to increase reliability, developed measures and cost estimates using robust procedures and included as an outcome the development of a QI project. While the findings from this study may not be directly transferable to other institutions, due to the small number of organisations participating on iQUASER, some of the general learnings, including the importance of the support of the Chair and CEO for the development of an organisation-wide QI strategy, may be applicable in a wider health care context. This is also similar with what other studies have found.^20,22,23^ As a result, one could consider that due to the relative inexpensive and effective nature of interventions such as iQUASER, policy-makers and healthcare managers may feel inclined to fostering and promoting change in QI strategy development by means of this type of interventions.

## Conclusion

Evidence suggests that there is an association between board practices and the quality of care provided by the organisation.^6,7^ However, few studies have demonstrated the impact of board-level interventions on QI.^6,7,9,24,25^ To our knowledge, this is the first cost-consequence analysis of a board-level intervention like iQUASER. We have found there was a positive association between level of engagement with the intervention, development of an organisation-wide QI strategy and the implementation of a QI project. More of the participating healthcare organisations improved in the development of an organisation-wide QI strategy than comparator organisations. We have also found that all participating organisations became more similar to the high-performing ‘benchmark,’ while fewer of the comparator organisations became more similar to this benchmark. Finally, we found that iQUASER, a board-level intervention for QI, is relatively inexpensive as a proportion of total NHS healthcare organisations’ budgets.

Although the evidence from this study is novel in that it not only considers consequences but also cost of a board-level QI intervention, more in-depth qualitative research is needed to fully understand our findings, particularly with regards to the mechanisms behind the improvements observed, which have been analysed elsewhere.^9^ Further work should also focus on more downstream process markers that might link to outcome measures, and explore the links between board-level interventions and these markers.

## Ethical issues

 This study was given exemption from NHS Research Ethics processes and oral informed consent for the interviews was obtained from all participants in accordance to NHS Research Ethics processes.

## Availability of data and material

 All data generated or analysed during this study are available from the corresponding author on reasonable request.

## Competing interests

 Authors declare that they have no competing interests.

## Authors’ contributions

 GB, SB, JEA, SM, and NF designed this study. LP and LJ were responsible for data collection. ECB, LP, and LJ have analysed the data. GB, SB, JEA, SM, and NF reviewed the analysis. ECB produced the first draft. All authors reviewed and approved the final manuscript.

## Endnotes


[1] NHS Foundation Trusts are semi-autonomous organisations that operate within the NHS in England. They have been described in detail by Robinson,^3^Walshe,^4^ Verzulli et al.^5^

[2] Quality account is a report about the quality of services provided by a NHS healthcare organisation. For more information about quality accounts, see https://www.nhs.uk/using-the-nhs/about-the-nhs/quality-accounts/.


## Authors’ affiliations

^1^Department of Applied Health Research, University College London, London, UK. ^2^University of Bangor, Bangor, UK. ^3^National Nursing Research Unit, Florence Nightingale School of Nursing and Midwifery, King’s College London, London, UK. ^4^Centre for Patient Safety and Service Quality, Faculty of Medicine, Imperial College London, London, UK. ^5^Florence Nightingale School of Nursing and Midwifery, King’s College London, London, UK. ^6^Department of Public Health and Primary Care, University of Cambridge, Cambridge, UK.

## Supplementary files


Supplementary file 1 contains Tables S1-S3.

Supplementary file 2. The iQUASER intervention.

Supplementary file 3. Implementation and evaluation of a research-based guide for boards of healthcare organisations to develop their quality improvement (QI)strategies: iQUASER.
