# Supplementary material for: A Board Level Intervention to Develop Organisation-Wide Quality Improvement Strategies: Cost-Consequences Analysis in 15 Healthcare Organisations
Source: Int J Health Policy Manag. 2020 Jun 28;11(2):173–82. doi: 10.34172/ijhpm.2020.91 (PMC9278604; doi:10.34172/ijhpm.2020.91)
Supplement: Supplementary file 1 — contains Tables S1-S3. [file ijhpm-11-173-s001.pdf]

Supplementary file 1

Table S1. iQUASER Attendance

	Workshop and ALS 1	ALS 2	ALS 3
Organisation 1	Chair<br>Director of Nursing<br>Chief Operating Officer<br>Non-Executive Director (Quality Lead)	Director of Nursing<br>Chief Operating Officer	Chief Operating Officer
Organisation 2	Chair<br>Medical Director<br>Chief Nurse	Medical Director<br>Director of Planning and Governance	Director of Planning and Governance
Organisation 3	Medical Director<br>Non-Executive Director (Quality lead)<br>Director of Quality and Safety	Medical Director<br>Medical Director (Quality)	Did not attend
Organisation 4	Director of Nursing<br>Clinical lead (nurse)<br>Medical Director<br>Non-Executive Director Associate Director of Governance	Medical Director<br>Associate Director of Governance	Associate Director of Governance
Organisation 5	Director of Nursing<br>Clinical lead (Consultant)<br>Non-Executive Director Non-Executive Director Non-Executive Director	Medical Director<br>Clinical lead	Medical Director<br>Clinical lead
Organisation 6	CEO<br>Director of Organisation and Transformation<br>NED	CEO<br>Director of Organisation and Transformation	CEO<br>Director of Organisation and Transformation

Abbreviations: CEO, chief executive officer; NED, non-executive director.

Table S2. Consequences Scores Before the Intervention and Level of Engagement

	Participating Organisations			Comparator Organisations						
Outstanding	Org 6 (CQC rating: Not assigned) Trust type: Acute	C1	3	Org 7 (CQC rating: Outstanding) Trust type: Acute and community	C1	3				
		C2	3		C2	3				
		C5	3		C5	-				
	Org 3 (CQC rating: not assigned) Trust type: Mental health	C1	1	Org 8 (CQC rating: Outstanding) Trust type: Mental health and community	C1	4		**Benchmark**		
		C2	2		C2	3		Org 13 (CQC rating: Outstanding) Trust type: Acute	C1	4
		C5	2		C5	-			C2	3
Requires Improvement	Org 5 (CQC rating: good) Trust type: Acute	C1	3	Org 9 (CQC rating: requires improvement) Trust type: Acute and community	C1	3				
		C2	2		C2	4				
		C5	3		C5	-				
	Org 1 (CQC rating: requires improvement) Trust type: Acute	C1	3	Org 10 (CQC rating: requires improvement) Trust type: Acute	C1	4		Benchmark		
		C2	2		C2	2		Org 14 (CQC rating: Requires improvement) Trust type: Acute and community	C1	1
		C5	1		C5	-			C2	2
Inadequate	Org 4 (CQC rating: Not assigned) Trust type: Acute	C1	1	Org 11 (CQC rating: Not assigned) Trust type: Acute and community	C1	4				
		C2	2		C2	3				
		C5	2		C5	-				
	Org 2 (CQC rating: requires improvement) Trust type: Acute	C1	4	Org 12 (CQC rating: Inadequate) Trust type: Acute	C1	2		Benchmark		
		C2	2		C2	2		Org 15 (CQC rating: Inadequate) Trust type: Community	C1	2
		C5	1		C5	-			C2	3

Abbreviation: CQC, Care Quality Commission.

Table S3. Consequences Scores After the Intervention

	Participating Organisations			Comparator Organisations						
Outstanding	Org 6 (CQC rating: Not assigned) Trust type: Acute	C1	4	Org 7 (CQC rating: Outstanding) Trust type: Acute and community	C1	3				
		C2	3		C2	2				
		C5	3		C5	-				
	Org 3 (CQC rating: not assigned) Trust type: Mental health	C1	2	Org 8 (CQC rating: Outstanding) Trust type: Mental health and community	C1	4		**Benchmark**		
		C2	2		C2	3		Org 13 (CQC rating: Outstanding) Trust type: Acute	C1	4
		C5	2		C5	-			C2	2
Requires Improvement	Org 5 (CQC rating: good) Trust type: Acute	C1	4	Org 9 (CQC rating: requires improvement) Trust type: Acute and community	C1	4				
		C2	3		C2	2				
		C5	3		C5	-				
	Org 1 (CQC rating: requires improvement) Trust type: Acute	C1	3	Org 10 (CQC rating: requires improvement) Trust type: Acute	C1	2		Benchmark		
		C2	2		C2	2		Org 14 (CQC rating: Requires improvement) Trust type: Acute and community	C1	1
		C5	1		C5	-			C2	2
Inadequate	Org 4 (CQC rating: Not assigned) Trust type: Acute	C1	2	Org 11 (CQC rating: Not assigned) Trust type: Acute and community	C1	4				
		C2	2		C2	2				
		C5	2		E	-				
	Org 2 (CQC rating: requires improvement) Trust type: Acute	C1	4	Org 12 (CQC rating: Inadequate) Trust type: Acute	C1	4		Benchmark		
		C2	2		C2	2		Org 15 (CQC rating: Inadequate) Trust type: community	C1	2
		C5	1		C5	-			C2	2

Abbreviation: CQC, Care Quality Commission.
