# Supplementary material for: A Board Level Intervention to Develop Organisation-Wide Quality Improvement Strategies: Cost-Consequences Analysis in 15 Healthcare Organisations
Source: Int J Health Policy Manag. 2020 Jun 28;11(2):173–82. doi: 10.34172/ijhpm.2020.91 (PMC9278604; doi:10.34172/ijhpm.2020.91)
Supplement: Supplementary file 2 — The iQUASER intervention. [file ijhpm-11-173-s002.pdf]

Supplementary file 2. The iQUASER Intervention

The intervention involved the following stages:

1. Individual organisational self-assessment using the QUASER Guide. Foresight Partnership (FP) created an on-line self-assessment survey based on the guide. This survey took approximately 30 minutes to complete. FP recommended that this was completed by the following individuals: All executive directors, including the chief executive, chair, non executive director with lead for quality, all other non-executive directors who wanted to take part, and, at the discretion of the board, divisional directors (clinical leaders) and/or those with a senior QI lead role not on the board. From the results of the self-completed assessment tool FP generated a report for each organisation to discuss and build a shared view of where the key quality improvement challenges are for each organisation. On the basis of the results of the self-assessment questionnaire each organisation was asked to:

i. Identify the areas of quality improvement that they feel need particular attention in their organisation

ii. Select one whole-organisation quality improvement intervention to pursue over the year of this programme

iii. Nominate three organisational leaders to participate in an initial workshop and action learning event, and two follow-up action learning events at 4 and 8 months. It was recommended that this included one executive director and one clinical leader. It was also suggested that participants may also include the NED who chairs the organisation's quality-focused subcommittee.

2. QUASER Workshop and Action Learning. This was a one-day workshop attended by senior leaders from all the participating organisations. The focus for the morning session was on the research that underpins the QUASER guide [1,2] and on developing a collective understanding of the QUASER Guide as a framework for developing elements of an organisation's quality

improvement strategy. The afternoon session was the first of three ‘action learning sets’. Two executive members of each participating organisation were allocated to small groups. The intention was that membership of these groups would remain constant throughout the programme.

The aim of the first action learning set was to create a supportive environment and for participating organisations to draw on the collective experience and wisdom of all the participants so as to:

i. Further develop an understanding of what approaches will be most effective to build an organisation-wide quality improvement strategy, based on each organisation’s current stage of development, and addressing the areas of quality improvement that may need most attention from their self-assessment.

ii. To explore and develop approaches to implementing an organisation-wide quality improvement intervention.

By the end of the day it was anticipated that all participants would have arrived at specific goals or commitments to take their quality improvement strategies and interventions forward.

3. Follow Up Action Learning Sets. Two follow-up facilitated action learning sets (at 3 and 6 months) explored implementation challenges, and ways to overcome them, as well as following up on progress. In addition the final learning set asked participants to reflect on the value of the guide in facilitating QI, as well as the value of facilitated support for implementation.

References

1. Burnett S, Mendel P, Nunes F, Wiig S, van Bovenkamp H, Karltun A, Robert G, Anderson J, Vincent C, Fulop NJ. Using Institutional Theory to Analyse Hospital Responses to External Demands for Finance and Quality in Five European Countries. *J Health Services Research & Policy* 2016; 21 (2): 109-117. doi:10.1177/1355819615622655.

2. Wiig S, Aase K, von Plessen C, Burnett S, Nunes F, Weggelaar M, Anderson-Gare B, Calltorp J, Fulop N. Talking about quality: exploring how 'quality' is conceptualized in European hospitals and healthcare systems. *BMC Health Services Research* 2014, 14:478. DOI: 10.1186/1472-6963-14-478
