# Supplementary material for: A Board Level Intervention to Develop Organisation-Wide Quality Improvement Strategies: Cost-Consequences Analysis in 15 Healthcare Organisations
Source: Int J Health Policy Manag. 2020 Jun 28;11(2):173–82. doi: 10.34172/ijhpm.2020.91 (PMC9278604; doi:10.34172/ijhpm.2020.91)
Supplement: Supplementary file 3 — Implementation and evaluation of a research-based guide for boards of healthcare organisations to develop their quality improvement (QI)strategies: iQUASER. [file ijhpm-11-173-s003.pdf]

Supplementary file 3. Implementation and evaluation of a research-based guide for boards of healthcare organisations to develop their quality improvement (QI) strategies: iQUASER

Interview outline

1. Please tell me about your role on the board/responsibilities.
2. How long have you been on the board?
3. How many other board positions have you held in the past?

Quality approach/Journey **(Educational/Physical & Tech/Structural)**

4. Can you tell me how you see the trust's overall quality improvement strategy?
5. To what extent do you think your Trust has an organisation-wide QI strategy (before and after) – on the scale below?
6. Do you think this scale is representative of where a Trust should be progressing toward/aiming for?

Impact Variables	Scales (Before and After)	Scales (Before and After)2	Scales (Before and After)3	Scales (Before and After)4
	**1**	**2**	**3**	**4**
**1. Organisation wide strategy**	1 (Very fragmented)	2 (Moderately fragmented)	3 (Managed Internally)	4 (One overall QI strategy document)

7. What is the nature of the discussions at board level about quality improvement in the trust? For example is sufficient time spent on this and do you have enough information for an informed discussion.

External/local **(Leadership/Political/External demands)**

8. How does the trust manage its internal quality improvement efforts whilst aligning with external demands (from e.g. commissioners, regulators)?

Top down/bottom up **(Leadership)**

9. Which quality improvement initiatives in your hospital would you classify as either driven from the board or driven by front line staff? Do you have the right balance and if not, why?

QI in relation to staff **(Leadership/Political/Cultural/Educational/Emotional/Physical & Tech/Structural)**

10. To what extent does your trust identify quality improvement priorities with your patients? How are these taken forward?

QI in relation to staff – values and beliefs **(Emotional/Cultural)**

11. Does the trust provide your staff with the opportunity to reflect on what 'quality' means, share their different understandings and increase awareness of different professional's perspectives?

Education in relation to QI **(Education/Structural)**

12. What quality improvement training is available to staff within the trust (provided either internally or by external agencies)?

Implementation **(Leadership)**

13. To what extent does your trust focus on implementation of long term strategies to improve the quality of care it provides? Can you give examples of any/some of these?

Use of data for QI **(Physical and Tech)**

14. How is QI measured and monitored in your trust (e.g. service-level dashboards) – what data are used?

iQUASER Questions

15. The changes you have spoken about (in response to the questions in yellow above) to what extent or not do you think these were influenced by the iQUASER Guide?
16. If they were influenced to some extent, how?
17. The changes you have spoken about (in response to the questions in yellow above) to what extent or not do you think these were influenced by the facilitated aspect of the intervention?
18. If they were influenced to some extent, how?
19. Did the iQUASER project deliver what you expected or not?
20. What would you change about this type of intervention if you were to sign up again?

Scale Questions

21. What do you think of the 8 challenge representation of the various components of the QI strategy?
22. To what extent do you think each of the challenges should be addressed by an organization?
23. How interconnected do you think the 8 challenges are/should be?

24. To what extent do you think your Trust is addressing or paying attention to each of the iQUASER challenges – on the following scale?

Impact Variables	Scales (Before and After)	Scales (Before and After)2	Scales (Before and After)3	Scales (Before and After)4	Scales (Before and After)5
	**1**	**2**	**3**	**4**	**5**
**2. Extent of addressing each challenge and level of maturity**	1 (No evidence of awareness)	2 (Planning)	3 (Doing)	4 (Evaluating an action undertaken)	5 (Revision or wider learning)

25) To what extent do you think the eight challenges are interconnected in your QI strategy – on the following scale?

Impact Variables	Scales (Before and After)	Scales (Before and After)2	Scales (Before and After)3	Scales (Before and After)4
	**1**	**2**	**3**	**4**
**3. Interconnectedness of the 8 challenges**	1 (Minimally connected)	2 (Moderately connected)	3 (Well connected)	4 (Maximally connected)

26) To what extent do you think the 8 challenges are balanced i.e. which challenges are most prominent in your Trust – on the following scale?

Impact Variables	Scales (Before and After)	Scales (Before and After)2	Scales (Before and After)3	Scales (Before and After)4
	**1**	**2**	**3**	**4**
**4. Balance of the 8 challenges**	1 (Full centrality)	2 (High centrality)	3 (Moderate centrality)	4 (Low centrality)

27) How much benchmarking with similar organizations does the trust do in terms of quality improvement?

28) How far (on the following scale) do you think the actions proposed as a result of the intervention are implemented (ie, the project)?

Impact Variables	Scales (Before and After)	Scales (Before and After)2	Scales (Before and After)3	Scales (Before and After)4
	**1**	**2**	**3**	**4**

**6. Implementation of the change actions**	1 (Not at all)	2 (To a little extent)	3 (To a moderate extent)	4 (To a large extent)

29) What other aspects do you think should be included in a measure that looks at an organisation's QI strategic maturity?

Cost Consequence

Did you spend any time on the iQUASER intervention?

How much time would you estimate you spent on the iQUASER intervention (QI strategy development and preparation for ALS etc)?

Conclusion

30) Confirm the implementation project the Trust is pursuing that we can follow up on over the next 12 months. What is the best way of following up the implementation of it – interviews, observation of meetings?

How much time has been spent on this to date?
When start working on it?
How many people working on it/who?
How much time would you estimate they are working on it?

31) When do you think the best time would be for us to hold the feedback seminar day we discussed at the final ALS? Nov 15, Jan 16, April 16?

32) We would like to gain the perspective of your service users – is there a patient body/governors that we could arrange to speak with?

33) Are there any views/perspectives on QI or the intervention itself that you don't feel you have been able to express in response to any of the other questions?
